# Regional infusion of a class C TLR9 agonist enhances liver tumor microenvironment reprogramming and MDSC reduction to improve responsiveness to systemic checkpoint inhibition

**DOI:** 10.1038/s41417-022-00484-z

**Published:** 2022-06-14

**Authors:** Chandra C. Ghosh, Kara R. Heatherton, Kyle P. O’ Connell, Ian S. Alexander, Deborah A. Greer, Jason LaPorte, Prajna Guha, Bryan F. Cox, Steven C. Katz

**Affiliations:** 1 Roger Williams Medical Center, Immuno-oncology Institute, Providence, RI USA; 2 TriSalus™ Life Sciences, Inc., Westminster, CO USA; 3 Northwestern University, Evanston, IL USA; 4 Department of Surgery, Boston University Medical Center, Boston, MA USA; 5 Department of Medicine, Roger Williams Medical Center, Providence, RI USA

**Keywords:** Tumour immunology, Immunosuppression

## Abstract

Myeloid-derived suppressor cells (MDSCs) expand in response to malignancy and suppress responsiveness to immunotherapy, including checkpoint inhibitors (CPIs). Within the liver, MDSCs have unique immunosuppressive features. While TLR9 agonists have shown promising activities in enhancing CPI responsiveness in superficial tumors amenable to direct needle injection, clinical success for liver tumors with TLR9 agonists has been limited by delivery challenges. Here, we report that regional intravascular infusion of ODN2395 into mice with liver metastasis (LM) partially eliminated liver MDSCs and reprogrammed residual MDSC. TLR9 agonist regional infusion also induced an increase in the M1/M2 macrophage ratio. Enhanced TLR9 signaling was demonstrated by an increased activation of in NFκB (pP65) and production of IL6 compared with systemic infusion. Further, PBMC-derived human MDSCs express TLR9, and treatment with class C TLR9 agonists (ODN2395 and SD101) reduced the expansion of MDSC population. TLR9 stimulation induced MDSC apoptosis and increased the M1/M2 macrophage ratio. Regional TLR9 agonist infusion along with systemic anti-PD-1 therapy improved control of LM. With effective delivery, TLR9 agonists have the potential to favorably reprogram the liver TME through reduction of MDSCs and favorable macrophage polarization, which may improve responsiveness to systemic CPI therapy.

## Introduction

CPI therapy has resulted in improved clinical outcomes across multiple indications, with notably less success in liver tumors. Indeed, the presence of LM is an independent predictor of CPI failure in cutaneous melanoma and non-small cell lung cancer, among other indications [1]. CPIs have also limited efficacy in uveal melanoma (UM), where metastatic spread is predominantly to the liver [2]. While up to 50% of UM patients eventually develop metastatic disease, more than 90% of these metastases form in the liver, and most of these patients survive less than a year [3–5]. The intrahepatic space drives immunosuppressive myeloid cell programming, which is exacerbated in the setting of malignancy [6–9]. The presence of LM is associated with systemic immune dysfunction and expansion of suppressive myeloid cells, which contribute to poor CPI clinical performance [1]. Novel combinatorial immunotherapy strategies are being exploited to improve clinical outcomes in patients with liver tumors, with an emphasis on addressing the uniquely suppressive intrahepatic milieu [7, 10, 11]. Given the distinctive immunologic features of the liver, appropriately tailored tumor microenvironment (TME) reprogramming strategies are needed.

Toll-like receptors (TLRs) play a key role in activating innate immunity by recognizing highly conserved molecules expressed by pathogens. Pattern-recognition receptors, such as TLR9, activate the innate immune system through recognition of small molecular motifs known as pathogen-associated molecular patterns [12]. TLR9 is known to be expressed in early endosomal compartments and bind to unmethylated bacterial CpG-DNA to activate immune cells [13]. TLR9 is expressed by a variety of cell types including B cells, dendritic cells, natural killer (NK) cells, and keratinocytes [14, 15]. It is generally believed that TLR9 is localized in the endosomal compartment [16], although some evidence suggest surface expression under a variety of conditions [17]. Murine MDSCs expresses TLRs such as TLR 2, 3, 4, 7, 8, and 9, but the expression of TLR9 on peripheral and liver MDSCs, is poorly defined [18, 19].

Three classes of TLR9 agonists define different target cells and varying immune reprogramming impact. Class A TLR9 agonists stimulate pDCs to enhance the production of IFNα, induce antigen presenting cells (APCs) and activate NK cells. Class B TLR9 agonists strongly activate B cells and NK cells with low IFNα induction. Class C TLR9 agonists have a broader immunologic effect, by stimulating pDC IFNα expression, APC activation and maturation, direct B cell activation, and indirect NK cell stimulation [20, 21]. Given the multiple non-redundant mechanisms of immunosuppression within liver TMEs, broader immune modulation may be more effective in patients with LM.

Synthetic class C TLR9 agonists such as ODN2395 and SD101 are structurally modified to make them nuclease resistant and increase their half-life [22]. In preclinical and clinical studies, TLR9 agonists used either as monotherapy or in combination therapy with immune checkpoint inhibitors showed anti-tumor responses [14, 15]. In general, TLR9 agonist monotherapy has not been clinically efficacious for solid tumors [14, 23]. Direct intralesional needle injection of a class B TLR9 agonists in combination with CPI did not result in clinical benefit [24]. In indications involving deep visceral tumors, which may be multifocal such as with UM and LM, locoregional intravascular delivery of TLR9 agonists may improve clinical performance of TLR9 agonists by enabling access to more immune cells in malignant lesions and surrounding parenchyma.

SD101 has been found to be well tolerated in combination with CPI across multiple indications [14]. Moreover, intra-tumoral injection of SD101 along with low dose radiation activated local immune response and induced an abscopal effect [25]. Combinatorial use of intra-tumoral SD101 with systemic anti-PD-1 checkpoint inhibitor for superficial tumors demonstrated that the combination was well tolerated with a response rate of 78% in CPI-naïve patients, and enhanced infiltrating lymphocytes in the TME [26]. The application of SD101 and other TLR9 agonists for liver tumors has been limited by the impracticality of direct needle injection and elevated intra-tumoral pressure, which may be overcome with innovative delivery technologies [27].

MDSCs inhibit proliferation of anti-tumor immune cells such as T cells and NK cells in the TME and promote tumor invasiveness [11, 28, 29]. We have recently shown that liver metastasis-derived MDSCs (LM-MDSCs) limit anti-tumor immunity predominantly via a STAT3-mediated mechanism, driven in part by GM-CSF [9]. MDSCs can differentiate into anti-tumorigenic M1 or pro-tumorigenic M2 macrophages, depending on the local TME [30]. Overall, LM-MDSCs display unique features relative to other sites. The present study was designed to test the hypothesis that regional intravascular infusion of class C TLR9 agonists can reprogram the LM-MDSC compartment to create a more immune-responsive TME. We demonstrate that regional infusion of a class C TLR9 agonist reduced MDSC abundance in LM and enhanced the antitumor efficacy of systemic anti-PD-1 therapy. These data expand our understanding of how class C TLR9 agonist biologic activity may be enhanced in the liver through innovative delivery approaches, allowing for critical drivers of intrahepatic immunosuppression to be addressed. If clinically validated, this approach may lay the foundation for improved clinical outcomes with systemic CPIs in patients with liver tumors.

## Materials/subjects and methods

### Mice, in vivo model, and treatment

C57BL/6 J (stock number:000664), aged 7–10 weeks male mice, were obtained from Jackson Laboratories (Bar Harbor, ME) and housed under pathogen-free conditions in the animal care facility at Roger Williams Medical Center (RWMC). All surgical procedures were performed as per RWMC Institutional Animal Care and Use Committee approved protocols. LM was generated by injecting 2.5 × 10^6^ MC38-CEA Luc cells (generous gift from Dr. Jeffrey Schlomm) via the spleen, followed by splenectomy as described previously [31]. MC38-CEA was tested for mycoplasma prior to use. In vivo bioluminescence imaging was performed by using IVIS Lumina II Imaging System to monitor tumor burden on D0, D1, and D2 as described before [7]. Mice were randomized into treatment groups so that animals in each group had a similar tumor burden. After 7 days (D0), mice were treated with 1, 3, 10, or 30 µg/mouse of ODN2395 via PV or 30 µg/mouse of ODN2395 via TV. PV infusions were done with the Pressure Enabled Drug Delivery™ (PEDD™) infusion model for enhanced flow and delivery pressure, as previously described [7]. Mice treated with PBS via PV were used as control. Mice were sacrificed on D2, and livers were harvested. Liver non-parenchymal cells (NPCs) were isolated, and CD45^+^ cells were purified using immuno-magnetic beads (Miltenyi Biotech, Cambridge, MA) as described previously [8, 31]. To evaluate combinatorial effect of CPI and ODN2395, LM-bearing mice received 250 µg/mouse of anti-mouse PD-1 antibody (Clone: RMP1-14, Bio X Cell, Lebanon, NH) intraperitoneally (IP) on D0, D3 and D10 and 30 µg/mouse ODN2395 via PV on D0. The number of mice used for each experiment was determined using G Power software and experimental replicates (biological and/or technical) are mentioned in respective figure legends. Mice were excluded from study if tumors were not generated or were sub-optimal (<10^6^ photons/s) as determined by in vivo bioluminescence imaging.

### Antibodies for western blotting, flow cytometry, and immunofluorescence

Antibodies and their corresponding clones used for flow cytometry (FC): anti-human CD11b (M1/70), CD33 (WM53), HLA-DR (G46-6), CD14 (M5E2), and CD15 (HI98) were obtained from BD Biosciences (Woburn, MA). Zombie NIR was used as a cell viability dye and CD86 (BU63) was obtained from (BioLegend, San Diego, CA). FC antibodies used to detect mouse MDSCs and macrophages were Gr1 (Ly6G/Ly6C, RB6-8C5), CD11b (M1/170), Ly6C (AL-21), and Ly6G (1A8) obtained from BD Biosciences, F/480 (BM8), CD38 (90) obtained from BioLegend, and EGR2 (ERONGR2) procured from Thermo Fisher Scientific (Waltham, MA). Antibodies against TLR7 (D7), TLR9 (D2C9), IL6 (D3K2N), phospho-NFκB p65^Ser536^ (93H1), total NFκB (C22B4), phospho-STAT3^Tyr705^ (D3A7) and total STAT3 (D3Z26) were obtained from Cell Signaling Technology (Danvers, MA). GAPDH (D16H11) was used for loading control and was obtained from Cell Signaling Technology.

### Protein analyses

Tissue samples from human patient LM biopsies were obtained from the RWMC Tissue Bank Core and the study was approved by the Institutional Review Board and patient’s informed consent was obtained. Harvested mouse liver lysates were used in western blotting (WB) as described previously [8, 31]. Samples were washed twice with ice-cold PBS and lysed with RIPA buffer (Thermo Fisher Scientific) in the presence of protease inhibitor cocktail (Roche Diagnostics, Indianapolis, IN). Samples were homogenized using porcelain beads as per manufacturer’s protocol (Thermo Fisher Scientific). Sonicated samples were then centrifuged at 10,000 rpm for 10 min at 4°C and the supernatant was collected. Protein quantification was performed using the Bradford protein assay (Thermo Fisher Scientific) using BSA (Sigma-Aldrich, St. Louis, MO) as the standard, per the manufacturer’s protocol. Lysates were denatured using Laemmli sample buffer (Bio-Rad, Hercules, CA) with β-mercaptoethanol (Thermo Fisher Scientific) and denatured by heating the samples at 95 °C for 5 min. Electrophoresis was performed using Mini Protean TGX 4–15% gels (Bio-Rad) and transferred on Trans-Blot Turbo PVDF membrane (Bio-Rad).

Cell supernatants obtained from in vitro experiments were tested for IL6, IL10, IL29, and IFNα using Procartaplex Luminex kit (Thermo Fisher Scientific) and measured by Magpix, (Luminex corp, Austin, TX). For immunofluorescence (IF), huPBMC, isolated from healthy donors from Rhode Island Blood Center as described previously [27], were grown in chamber slides (Millipore, Burlington, MA). After fixing, cells were blocked and incubated with primary antibodies (1:100) at 37 °C for 1 h. Secondary antibodies (1:250) conjugated with appropriate fluorophore were incubated at room temperature for 1 h. Secondary antibody-only incubated samples served as negative controls for the procedure. Prolong-DAPI (Thermo Fisher Scientific) was used for nuclear staining. All images were captured using a Zeiss LSM 700 confocal laser-scanning microscope (Zeiss, Dublin, CA) at 63X magnification. For FC, 2.5 × 10^5^ cells incubated with antibodies for 30 min at room temperature (RT), stained with BioLegend Zombie NIR (human cells only) for 30 min at RT, fixed with Cytofix (BD Biosciences) and ran on a CytoFLEX LX flow cytometer (Beckman Coulter, Brea, CA). Compensation beads (Thermo Fisher Scientific) were used to set compensation and isotype controls were used to set gates. Flow cytometry data were analyzed by using CytExpert software (Beckman Coulter).

### Secreted embryonic alkaline phosphatase (SEAP) assay

For TLR9-dependent NFκB reporter assay, HEK293-Blue cells were used per as the manufacturer’s protocol (Invivogen, San Diego, CA). Cells were generated by co-transfecting the murine TLR9 gene and an inducible SEAP reporter gene into HEK293 cells. The SEAP gene was placed under the control of the interferon-beta (IFNβ) minimal promoter fused to five NFκB and activator protein-1 (AP-1) binding sites. Stimulation with a TLR9 ligand activates NFκB and AP-1, which induce the production of SEAP and are measured by a plate reader at 650 nm. Cells were treated with ODN2395 and SD101 at increasing doses (0.004–10 µM) for 21 h. As a negative sequence control for ODN2395 ODN5328 (C) (Invivogen, San Diego, CA) was used. Sequence control contains GpC dinucleotides instead of CpG present in ODN2395 (Invivogen, San Diego, CA).

### qRT-PCR

Total RNA was isolated from tissues and cells using RNeasy Mini Kit (Qiagen, Germantown, MD). iScript cDNA Synthesis kit (Bio-Rad) was used for reverse transcription and SYBR Master Mix (Bio-Rad) was used for quantitative PCR. Primers for human TLR9 was obtained from Bio-Rad (proprietory).

The primers used were as follows:

**Table:** 

Gene	Species	Forward	Reverse
TLR9	Mouse	ATGGTTCTCCGTCGAAGGACT	GAGGCTTCAGCTCACAGGG
IL10	Mouse	GCTCTTACTGACTGGCATGAG	CGCAGCTCTAGGAGCATGTG
GAPDH	Mouse	GGCATTGCTCTCAATGACAA	ATGTAGGCCATGAGGTCCAC
RPL27	Human	GTGGCTGGAATTGACCGCTA	ACAGAGTACCTTGTGGGCATT

For all samples, ΔCt values were calculated, and RPL27 (human) or GAPDH (mouse) were used to normalize the gene expression.

### Statistics

The investigator was not blinded to the experimental group during data collection and analysis. Statistical significance was performed using students’ *t* test, multiple *t* test and one-way ANOVA. For multiple *t* test, discovery was determined using the two-stage linear step-up procedure of Benjamini, Krieger, and Yekutieli, with *Q* = 1%. Each row was analyzed individually without assuming a consistent SD. Post-hoc multiple comparison test was performed when ANOVA was significant. Prism (V8) software (Graphpad, San Diego, CA) was used to analyze data. For all studies, values of *p* < 0.05 were considered statistically significant. G Power software was used to determine sample size for each experiment.

## Results

### Regional delivery of TLR9 agonist via PV inhibits the progression of LM

**Fig. 1:**
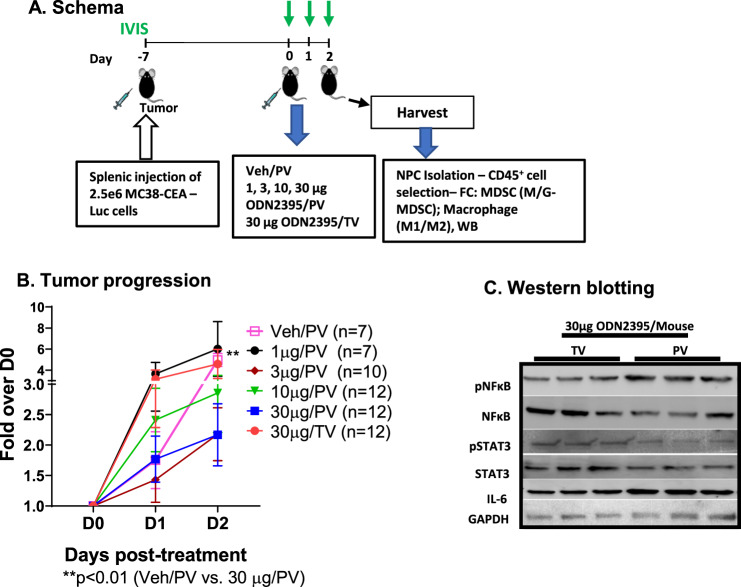
ODN2395 administered via PV is more effective in inhibiting tumor progression. **A**. Schema: Schematic representation of the timeline of LM generation and the treatment protocol. Eight- to twelve-week-old C57/BL6 mice were challenged using intra-splenic injection model with 2.5 × 10^6^ MC38-CEA-Luc cells for 7 days (D-7). Bioluminescence value was determined by IVIS on D0, D1, D2, and mice were randomized accordingly and treated with 1, 3, 10, or 30 µg/mouse ODN2395 via portal vein (PV) and 30 µg/mouse ODN2395 via tail vein (TV). PBS served as the vehicle (Veh) control and administered via PV. On D2 post-treatment with ODN2395 or Veh, mice were sacrificed, and liver was harvested to isolate CD45^+^ cells. Isolated CD45^+^ NPCs were evaluated for MDSCs and macrophages (M1 and M2). **B** Tumor progression was monitored by IVIS imaging on the day of treatment (D0), D1, and D2 post-treatment. Fold change of the tumor burden was calculated based on D0 baseline bioluminescence. Multiple t test was performed to determine the significant difference. **C** Harvested LM tissues (whole lysates) from *n* = 6 mice/group (representative of *n* = 3 shown) were evaluated for pNFκB (p65^S536^), pSTAT3^Y705^, total NFκB, STAT3, and IL6 by western blotting. GAPDH was used as a housekeeping protein control.

To evaluate single-agent activity of a class C TLR9 agonist delivered by regional intravascular infusion using the PEDD™ murine model to enhance flow and pressure, we treated LM-bearing mice with 1, 3, 10, or 30 μg of class C ODN2395 via PV or TV (30 μg) per the schema (Fig. 1A). Bioluminescence was measured at baseline, 24-, and 48-h post-ODN2395 administration to quantify LM burden. We found that 30 μg ODN2395 via PV had significantly improved tumor killing compared to vehicle control at D2 (bioluminescence fold change = 1.08 vs. 2.80; *p* < 0.01) (Fig. 1B), whereas TV infusion did not improve tumor control. There was a non-significant trend towards decreased tumor burden with 3 μg (D1; *p* = 0.10; D2; *p* = 0.14) and 30 μg ODN2395 (D1; *p* = 0.16; D2; *p* = 0.11) via PV as compared to 30 μg TV.

Class C ODNs activate both NFκB and IFNα pathways. We hypothesized that a TLR9 agonist delivered regionally would result in enhanced NFκB activation as compared to systemic administration. We harvested the LMs, performed WBs, and found that mice that received 30 μg ODN2395 via PV had enhanced pNFκB/NFκB (1.77 vs. 0.68; *p* < 0.01) ratio along with increased IL6 (2.7 vs. 0.8; *p* < 0.05) expression and reduced pSTAT3/STAT3 (1.066 vs. 0.3865; *p* < 0.05) ratio as compared to mice treated via TV (Fig. 1C, Supplementary Fig. 1)).

### Class C TLR9 agonists delivered via PV alter the immunosuppressive phenotype of myeloid cells and promote M1 macrophage polarization

**Fig. 2:**
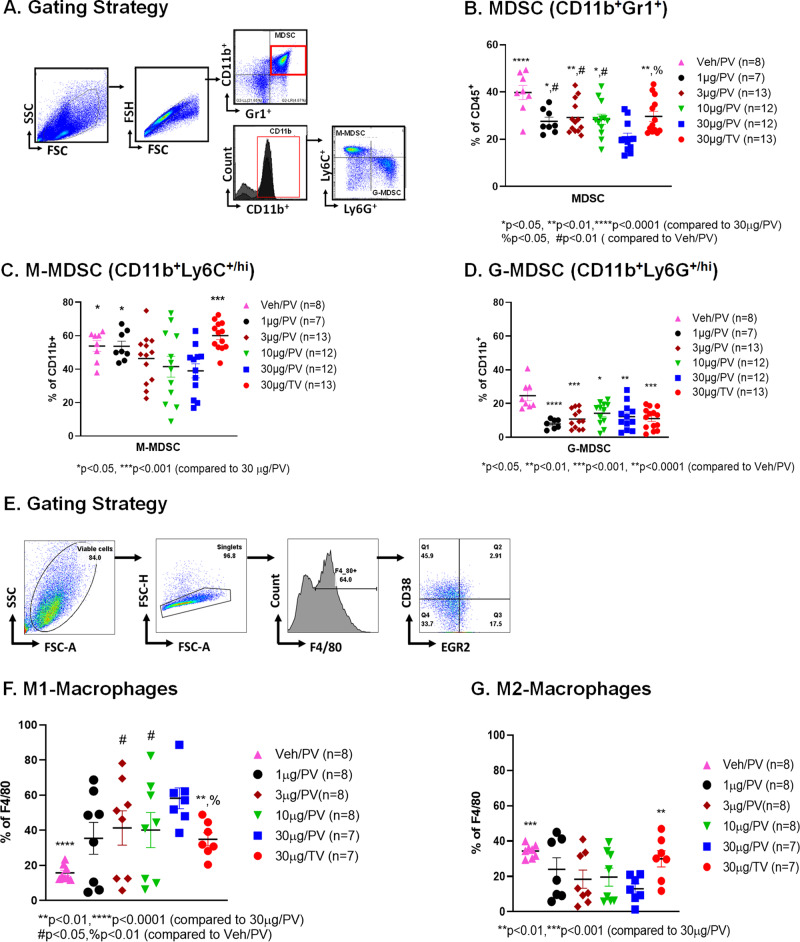
ODN2395 administered via PV reprograms the myeloid cell phenotype in the LM. **A** As described in Fig. 1A, mice were sacrificed after 2 days post-treatment. CD45^+^ cells were isolated from non-parenchymal cells (NPCs). Gating strategy to analyze CD45^+^ cells isolated from the LM. **B** MDSC cell population (CD11b^+^Gr1^+^), **C** monocytic MDSCs (M-MDSC; CD11b^+^Ly6C^+/hi^Ly6G^/lo^) and **D** granulocytic MDSCs (G-MDSC; CD11b^+^Ly6C^-/lo^Ly6G^+/hi^) were measured. **E** Gating strategy of the phenotypic analysis of CD45^+^ derived macrophages that were isolated from the LM. **F** M1-macrophage cell population (F4/80^+^CD38^+^Egr2^-^) and **G** M2 macrophage cell population (F4/80^+^CD38^-^Egr2^+^) were determined. Each animal data is represented by a scattered plot and presented as mean ± SEM from at least three different experiments. Students’ *t*-test was performed for group-wise comparison and are described in each graph.

To investigate the effect of class C TLR9 agonists on immunosuppressive LM-MDSC expansion, bulk hepatic NPCs from LM-bearing mice were enriched for CD45^+^ cells and the frequency of MDSCs was measured (Fig. 2A). Having previously shown that M-MDSC are the dominant suppressive subset in the liver, M-MDSC and G-MDSC were quantified separately [31]. Mice that received 30 μg ODN2395 via PV had a significantly reduced LM-MDSC population as compared to vehicle (Veh) control (20.75% vs. 39.78%; *p* < 0.0001) (Fig. 2B). Treating mice with 30 μg ODN2395 via PV was superior in reducing total MDSCs (20.75% vs. 29.70%; *p* < 0.01) and M-MDSCs (38.98% vs. 60.03%; *p* < 0.001) (Fig. 2C) as compared to the same dose via TV. In addition, low PV doses (10 μg and 3 μg) reduced M-MDSC relative to TV in a non-significant manner. The liver G-MDSC population was affected similarly by all doses and routes (Fig. 2D).

M2 (F4/80^+^CD38^-^Egr2^+^) macrophages, like MDSCs, are immunosuppressive, while M1 (F4/80^+^CD38^+^Egr2^-^) macrophages mediate anti-tumor immune responses. As determined by FC (Fig. 2E), mice treated with ODN2395 had a significantly increased M1 macrophage population (Fig. 2F) compared to the control group (*p* < 0.05), irrespective of the route of delivery except for 1 µg/PV group. However, liver M1 macrophage polarization was significantly increased when the class C TLR9 agonist was delivered via PV (58.20% 30 μg PV vs. 34.82%; *p* < 0.01 30 μg TV) in 30 μg ODN2395/PV compared to 30 µg/TV group. The M2 population was significantly reduced in mice treated with 30 μg ODN2395/PV compared to vehicle (12.99% vs. 29.96%; *p* < 0.01) and 30 µg/TV (12.99% vs. 34.30%; *p* < 0.0001) treated mice, as shown in Fig. 2G. Accordingly, 30 μg ODN2395, when delivered via PV, significantly increased M1/M2 ratio as compared to the TV group (*p* < 0.05). A non-significant trend towards increased CD3^+^ cells in ODN2395 + α-PD1 as compared to vehicle and α-PD1, was observed (data not shown).

### ODN2395 and SD101 activate NFκB signaling via TLR9 activation in a non-linear manner

**Fig. 3:**
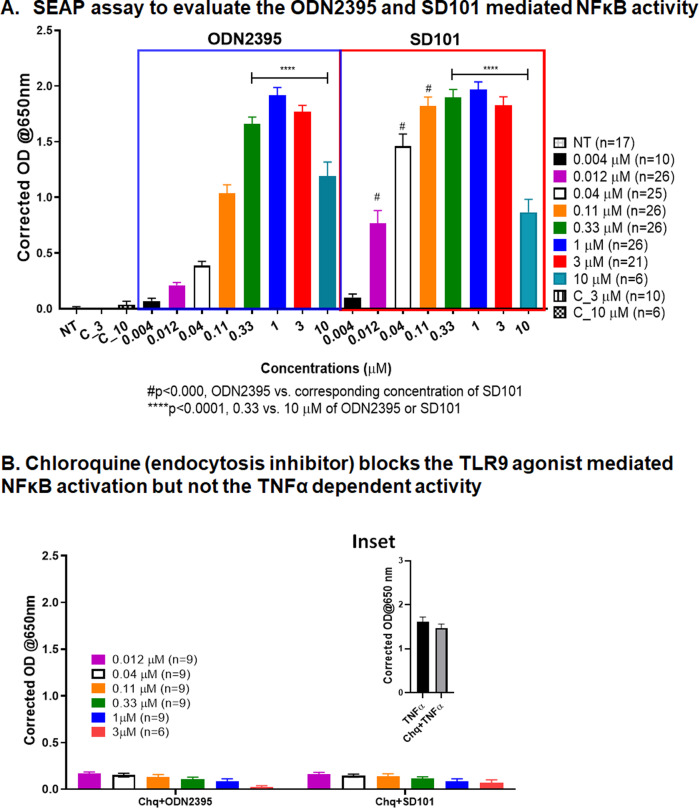
Determination of TLR9-dependent activity of TLR9 agonists. **A** In this reporter-based assay, TLR9-expressing HEK293-Blue cells were treated with ODN2395 and SD101 at increasing doses (0.004–10 µM) for 21 h. As a negative control, no-treatment (NT) and sequence control ODN5328 at 3 (C_3) and 10 (C_10) µM were used. The secreted embryonic alkaline phosphatase (SEAP) was determined by measuring the absorbance at 650 nm after addition of substrate. **B** Cells were pretreated with chloroquine (Chq, 1 µg/ml) for 45 min before the addition of ODN2395 or SD101 at increasing concentrations (0.012–3 µM) for 21 h, and absorbance was measured at 650 nm. All the experiments were performed at least three times with 2–3 replicates, and mean ± SEM was plotted in the graph.

Having demonstrated that regional intravascular delivery of a class C TLR9 agonist enhanced NFκB phosphorylation, we sought to compare the potency of ODN2395 with SD101, the latter being a class C TLR9 agonist presently under development for UM LM (NCT04935229). Similar non-linear dose-dependent responses were observed for both ODN2395 and SD101 with respect to TLR9 signaling activity (Fig. 3A). The negative sequence control ODN5328 at 3 and 10 µM as well as non-treated cells did not produce appreciable TLR9 activation (Fig. 3A). The requirement for TLR9 activity in activating NFκB was determined by pretreating cells with 1 μg/mL chloroquine (Chq), an inhibitor of endosomal maturation, prior to exposure to ODN2395 or SD-101. Figure 3B shows that Chq completely inhibited NFκB activation by ODN2395 and SD101-treated cells (0.012–3 μM). However, cells pretreated with Chq followed by canonical NFκB activation by tumor necrosis factor-alpha (TNFα; 20 ng/ml) stimulation did not affect SEAP production Fig. 3B (inset).

### Class C TLR9 agonists reduced human peripheral MDSC in vitro while enhancing PBMC NFκB- and IFNα-dependent cytokines

**Fig. 4:**
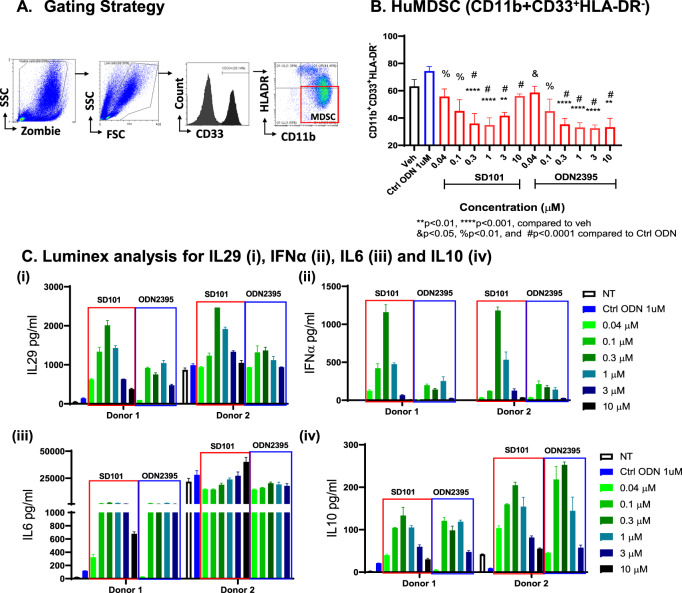
TLR9 agonist-treated huPBMC reduced MDSC population and increases IFNα- and NFκB-regulated genes. **A** Gating strategy for phenotypic analysis of MDSCs; isolated human PBMCs were treated with increasing concentrations (0.04–10 µM) of SD101, ODN2395 along with ctrl ODN5328 (1 µM) for 48 h. **B** MDSC population was quantified followed by FC analysis. Four donors with three replicates were used, and data represented as mean ± SEM (*n* = 12). **C** Supernatants of Donor 1 and 2 were collected analyzed for (i) IL29, (ii) IFNα, (iii) IL6, (iv) IL10 by using Luminex. Cells treated with SD101 and ODN2395 are represented as red and blue boxes, respectively. Data from two representative donors with two replicates are presented here. For Donor 1 and 2 supernatants from 10 µM ODN2395 treated samples were unavailable for luminex potential analysis.

To evaluate the effect of class C TLR9 agonists on huMDSC population (CD11b^+^CD33^+^HLA-DR^lo^), isolated PBMCs from healthy donors were treated with increasing concentrations of class C ODN2395 and SD101 for 48 h (Fig. 4A). We found that both SD101 and ODN2395 decreased the huMDSC population (Fig. 4B). However, the MDSC-diminishing effect decreased with increased concentrations (3 μM and 10 μM) of SD101. Further, 0.3 μM dose for both TLR9 agonists seemed to be optimal in decreasing the huMDSC population. The cell supernatants were analyzed by Luminex for IL6, IL10, IL29, and IFNα (Fig. 4C and Supplementary Fig. 2). All donors (*n* = 4) responded to the class C TLR9 agonists in a biphasic manner.The Luminex analysis performed on supernatant collected from cells treated (0.04–10 µM) of SD101, ODN2395 along with sequence ctrl ODN5328 (1 µM) for 48 h. For Donors 1 and 2, supernatants from 10 µM ODN2395 treated samples were unavailable for luminex analysis. Class C TLR9 agonist-mediated cytokine induction initiated at 6 h post treatment (data not shown). There was a donor-to-donor baseline variability in the cytokine production although they all had similar pattern in the cytokine production by huPBMCs treated with SD101 or ODN2395.

### TLR9 is expressed in human LM tissue and on the surface of huMDSCs

**Fig. 5:**
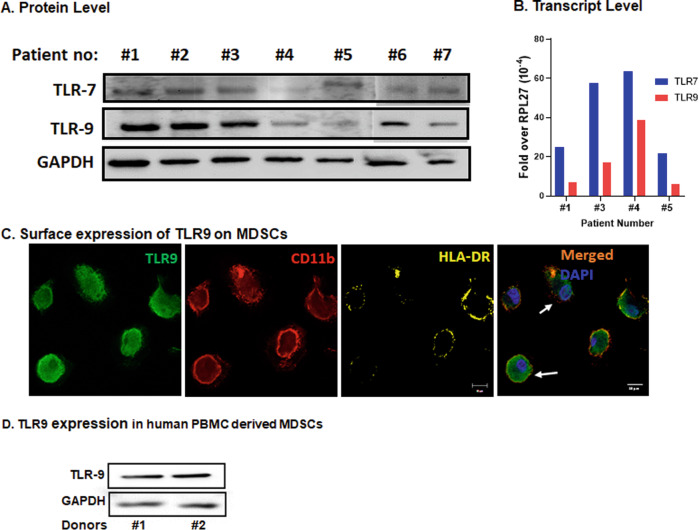
Expression of TLR9 in LM biospecimens and MDSCs. **A** Protein lysates obtained from LM patient biospecimens were evaluated for TLR7 and TLR9 by western blotting. GAPDH was used as housekeeping protein control. WB was performed on two different runs (#1 to #5 on one run and #6 and #7 were performed in a different run) **B** Total RNA was isolated from the same biospecimen and expression of TLR9 was quantified by qRT-PCR. RPL-27 gene was used as housekeeping control. Representative data of 4 of 7 biospecimens is shown here due to sample unavailability). **C** IL6 (20 ng/ml) + GMCSF (20 ng/ml) stimulated PBMCs grown in chamber slides were fixed and stained with TLR9 (green), CD11b (red) and HLA-DR (yellow) antibodies and DAPI (blue) used for nuclear staining. IF images demonstrate surface expression of TLR9 in CD11b^+^HLA-DR^−^ cells. Representative of three different experiment using PBMCs from three different donors. **D** WB of IL6 (20 ng/ml) + GMCSF (20 ng/ml) treated PBMCs showing expression of TLR9. GAPDH was used as control. (Representative of two out of four donors).

Preclinical murine data demonstrated that class C TLR9 agonist delivered via PV reduced LM burden, possibly by altering the TME and enabling anti-tumor immunity. Functional data confirmed that ODN2395 and SD101 mediated increase in pro-inflammatory cytokine is TLR9 dependent and decreased MDSC cell population in huPBMCs. We confirmed the expression of TLR9 and related endosomal protein TLR7 in the LM samples at protein and transcript levels on the tissues obtained from seven different cancer patients (Fig. 5A, B). Thus, the presence of TLR9 in human LM samples demonstrates the potential for regional delivery of a TLR9 agonist such as SD101 to recapitulate our preclinical murine efficacy when administered in a clinical setting.

TLR9 is predominantly expressed in the endosomal compartment. However, studies show that TLR9 is also expressed on the cell surface of splenic DCs, rat peritoneal mast cells, and in certain experimental settings [17]. Using IF, we confirmed that huMDSCs (CD11b^+^CD33^+^HLA-DR^lo/-^) express TLR9 on their surface (Fig. 5C). WB data on lysates obtained from IL6 (20 ng/ml) + GMCSF (20 ng/ml) treated huPBMCs further confirmed the expression of TLR9 in the MDSC-enriched cells (Fig. 5D). Further, qRT-PCR data of CD11b^+^Gr1^+^ magnetically beaded MDSCs from mouse LM also confirmed the expression of TLR9 transcripts (Supplementary Fig. 3), and SD101 did not alter the expression of TLR9 transcripts.

### Class C TLR9 agonists inhibit the differentiation of huMDSCs from huPBMCs

**Fig. 6:**
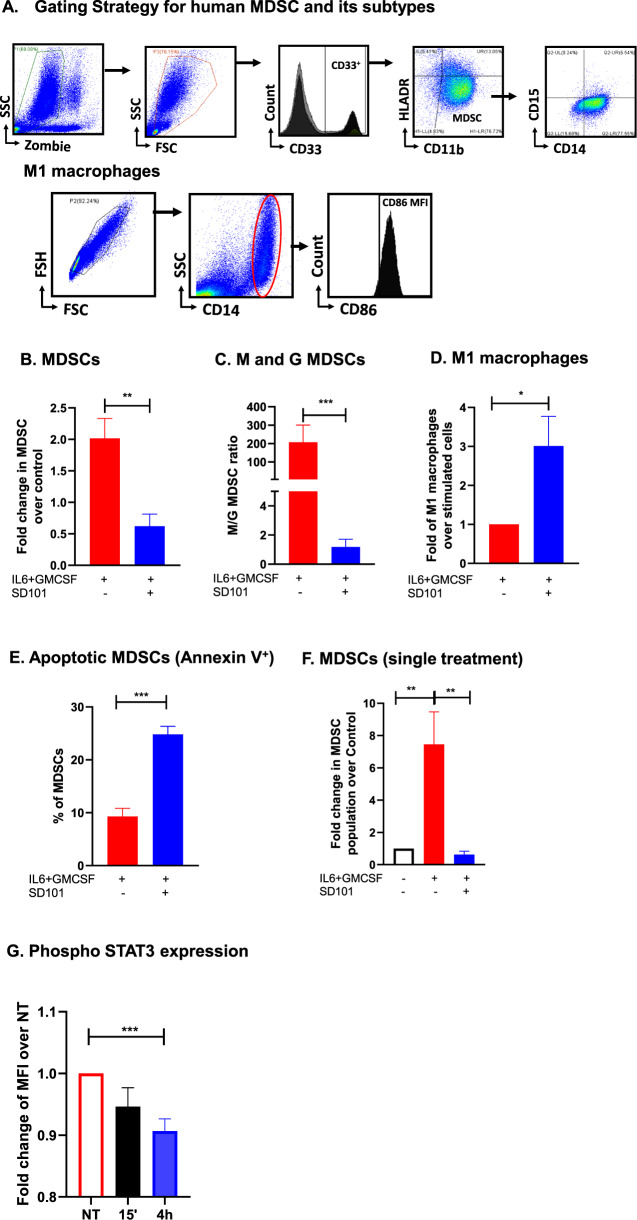
SD101 inhibits the generation of huMDSCs from PBMCs. **A** Gating strategy to identify huMDSCs, its subtypes M- and G-MDSCs and M1 macrophages. PBMCs were treated with IL6 (20 ng/ml) + GMCSF (20 ng/ml) for 7 days in the presence or absence of 0.3 µM SD101. **B** Cells were treated with SD101 on D0, D2, and D7 and percentage of MDSCs (CD11b^+^CD33^+^HLA-DR^lo/−^) was measured. **C** Ratio of M-/G-MDSCs was quantified; M-MDSCs: CD11b^+^CD14^+^CD15^-^HLA-DR^lo/−^, G-MDSCs:CD11b^+^CD14^-^CD15^+^HLA-DR^lo/−^. **D** Macrophage population was quantified: CD14^+^CD86^+^. **E** Annexin positive MDSCs were quantified. **F** PBMCs were treated once on D0 with SD101 (0.3 µM) for 48 h and MDSC was quantified. **G** PBMCs were stimulated with IL6 + GMCSF and treated with SD101 for 15 min and 4 h. FC analysis was performed to quantify pSTAT3 MFI in the MDSC gated cells and reported as fold change in the MFI of pSTAT3 positive cells. All the experiments were performed at least three times, and mean ± SEM was plotted in the graph.

To investigate the effect of SD101 on the differentiation of huMDSCs from PBMCs, we stimulated huPBMCs with IL6 (20 ng/ml) + GMCSF (20 ng/ml) and treated with SD101 for 7 days to induce the cytokine and growth factor induced MDSC transformation shown in and identified huMDSC as shown in Fig. 6A. SD101 treatment significantly reduced the huMDSC population (Fig. 6B). Furthermore, similar to the murine LM model, SD101 preferentially reduced the M-MDSC subset (Fig. 6C) and significantly increased (3-fold) M1 macrophage polarization (Fig. 6D). Further SD101 induced (9.28 vs. 24.81; *p* < 0.001) MDSC apoptosis as measured by Annexin V positive cells (Fig. 6E). A single treatment with SD101 was sufficient to inhibit huMDSC differentiation (Fig. 6F).

Increased phosphorylation of STAT3 is implicated in expansion of LM-MDSCs [8, 31]. We hypothesized that SD101 would inhibit the STAT3 phosphorylation of MDSCs, thereby inhibiting their expansion. We generated huMDSCs by treating huPBMCs with IL6 + GMCSF for 6 days. Enriched MDSCs on day 6 were treated with SD101 (0.3 µM) for 15 min or 4 h. FC analysis showed significantly reduced phosphorylation of STAT3 (*p* < 0.05) in cells treated with SD101 for 4 h as compared to NT group (Fig. 6G).

### Regional administration of a TLR9 agonist potentiated the responsiveness of anti-PD-1 therapy

**Fig. 7:**
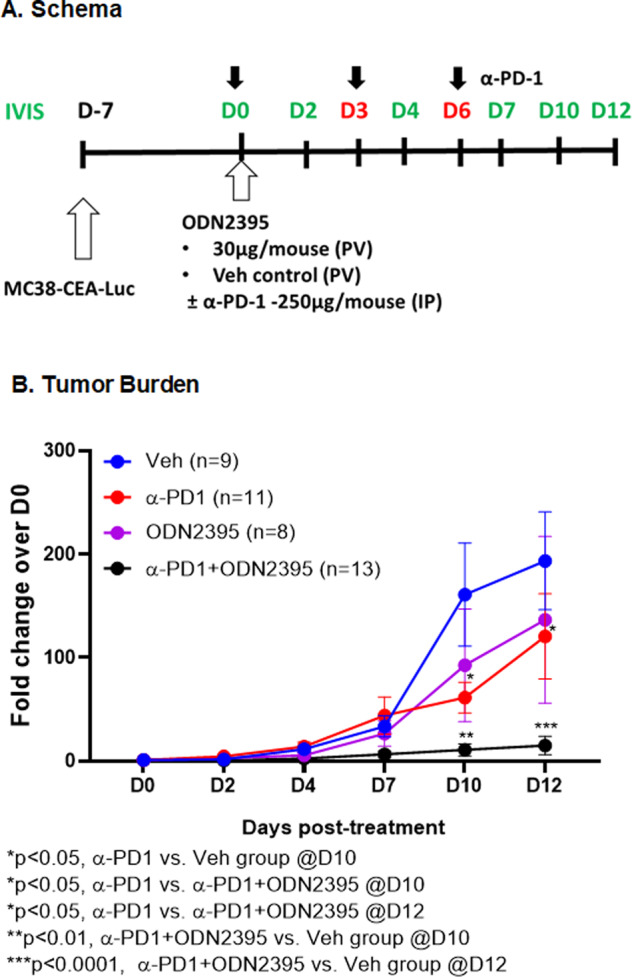
Locoregional TLR9 stimulation with systemic CPI effectively inhibits tumor progression. **A** Schema illustrates the steps to develop LM and the treatment regimen. Eight- to twelve-week-old male C57/BL6 mice were challenged using intra-splenic injection model with MC38-CEA-Luc cells for 1 week. Bioluminescence was measured by IVIS and mice were randomized on D0 prior to treatment with 30 µg/mouse ODN2395 via PV with or without 250 µg/mouse of anti-PD1 antibody via IP on D0, D3, and D6. PBS-treated mice via PV were used as control. **B** Tumor growth was monitored by IVIS imaging on D2, D4, D7, D10, and D12. Fold change of the tumor burden was calculated based on D0 baseline bioluminescence. Data was analyzed by multiple *t* test. Discovery determined using the two-stage linear step-up procedure of Benjamini, Krieger and Yekutieli, with *Q* = 1%.

Having shown that single-agent regional class C TLR9 agonist treatment was capable of LM control, efficient TLR9 activation, and MDSC elimination, we tested the impact on systemic CPI responsiveness to model an ongoing phase 1/1b study for UM LM (NCT04935229). Mice with established LM were treated with ODN2395 (30 µg/mouse) via PV with or without systemic anti-PD1 antibody (α-PD1: 250 µg/mouse) via IP as shown in Fig. 7A. Regional intravascular class C TLR9 stimulation significantly enhanced the ability of systemic CPI therapy to control LM burden compared to vehicle treated group (fold change over D0: 14.99 vs. 193.5 @D12; *p* < 0.001) as well as single-agent α-PD1 (fold change over D0: 14.99 vs. 120.4 @D12; *p* < 0.05) and ODN22395 (fold change over D0: 14.99 vs. 136.5 @D12; *p* = 0.08) (Fig. 7B). Combinatorial treatment initiated this antitumor effect beginning at D4 (fold change over D0: 2.49 vs. 11.45; *p* < 0.05).

## Discussion

Regional intravascular delivery of a class C TLR9 agonist in a murine model of PEDD™ enhanced control of LM, favorably reprogramed liver myeloid populations, and enabled systemic CPI. The effect of class C TLR9 agonist on MDSCs was confirmed in both the murine liver and with human PBMCs in vitro. While MDSCs are important drivers of intrahepatic immunosuppression and CPI failure, liver immune dysfunction is likely the result of a complex network of multiple factors. Fortunately, class C TLR9 agonists can stimulate both adaptive and innate immunity through multiple cell types to potentiate antitumor immune responses.

The liver is a unique organ that is intrinsically immunosuppressive due to the presence of suppressive cells such as MDSC and Tregs, in addition to cytokines secreted by these cells such as IL10 and TGFβ [32]. The intrahepatic space contains an abundance of MDSCs in the presence of tumors which are key drivers of the immunosuppressive TME. The extent of MDSC expansion is dependent on the tumor burden and the extent of the disease [33]. We have previously shown that MDSCs have the ability to adapt to organ-specific environmental cues, and when exposed to the intrahepatic space, adopt a specific molecular program while skewing toward the M-MDSC subtype [31]. Here, we have observed a decrease in total liver MDSCs and a relative reduction in M-MDSCs in mice with LM treated with a class C TLR9 agonist via PV. The suppressive nature of the liver itself and TMEs make regional intravascular infusion of a TLR9 agonist attractive in that immune cells throughout the organ and within all intrahepatic tumors may be treated.

TLR agonists when used in monotherapy or in combination with CPIs, especially when applied locally for superficial tumors or regionally for deep tumors, have demonstrated the potential to control tumor growth and mediate TME reprogramming effects [34–36]. TLR9 agonists have been used in preclinical and clinical platforms as a monotherapy or in combination with radiation, chemotherapy, targeted therapy, or immunotherapy [15, 37, 38]. While the present study did demonstrate monotherapy activity with a class C TLR9 agonist when delivered regionally, more profound control of LM was achieved when combined with systemic CPI. Our data demonstrate that regional TLR9 agonist infusions addressed a critical driver of intrahepatic immunosuppression by reducing liver MDSCs in association with STAT3 deactivation. Moreover, MDSC elimination was accompanied by favorable macrophage polarization. Based on prior reports, STAT3 deactivation induces liver MDSC apoptosis, which was demonstrated in the current study following regional class C TLR9 agonist infusion. STAT3-dependent liver MDSC expansion and suppressive programming are important drivers of MDSC-mediated liver immune dysfunction [8, 9, 31, 32].

The effects of regional class C TLR9 agonist treatments were not limited to MDSC within the liver. M1 macrophages can be activated by TLR agonists and support antitumor immunity. In contrast, M2 macrophages promote immunosuppression and pro-tumorigenic activities [39]. Plasticity of macrophages is dependent on multiple signals in the TME and the polarization state at any given point in time is not fixed [8, 31, 39]. We have previously shown that there is signaling-dependent fluidity among macrophage phenotypic states which is organ dependent [31]. In this study, we have shown that class C TLR9 agonists can drive immunogenic polarization of macrophages through increased M1/M2 ratios, supporting a more pro-inflammatory and anti-tumorigenic TME.

Activation of both the NFκB and STAT3 pathways enhance the expansion and accumulation of MDSCs in the tumor [40, 41]. In this study, we observed enhanced pNFκB and IL6 signaling with reduced pSTAT3 activation within LM following class C TLR9 agonist treatment via PV. STAT3 has a role in tumor immunity by promoting pro-oncogenic inflammatory pathways, including nuclear factor-κB (NF-κB) and interleukin-6 (IL-6)–GP130–Janus kinase (JAK) pathways [42], however, the non-canonical NFκB pathway regulated STAT3 dependent MDSC activity needs further investigation[43–45]. We found a biphasic regulation of NFκB-dependent signaling for SD101 and ODN2395. Consistent with this, a preclinical study showed that SD101 induced IFNα and IL10 in huPBMC in a “bell shaped curve” dose response, suggesting high TLR9 stimulus may induce negative feedback [46]. Hence determining the optimal dosing of TLR9 agonist may be necessary.

Activation of the transcription factor NFκB could initiate anti- or pro-apoptotic signaling depending on the cell type where it is expressed [47]. For example, DNA-damaging agents such as daunorubicin and serum withdrawal from HEK293 cells or Sindbis-Virus-induced apoptosis in a carcinoma cell line all cause NFκB activation-induced apoptosis [48–50]. In this study, we found that SD101 induced apoptosis in the huMDSC population. To further understand the effect and underlying mechanism of class C TLR9 agonist on LM-MDSCs, similar experiments should be performed in TLR9^−/−^ mice [51]. A detailed genomic and proteomic landscape, particularly for MDSCs, could reveal novel mechanisms for monotherapy or combinatorial activity of class C TLR9 agonists in the liver.

CPIs have transformed cancer treatment, with unprecedented and durable anti-tumor response observed with antibodies against key immunological checkpoints such as PD-L1, PD-1, and CTLA-4 [52]. However, only certain cancer types have benefited from this treatment and treatment options are limited for CPI relapsed or refractory patients [53]. Growing evidence suggests that the TME can be tailored by direct intra-tumoral injections of immunostimulatory agents such as TLR9 agonists that can convert the “cold” tumors with low immune infiltrates to “hot” tumors with high immune infiltrates that are more responsive to CPI treatment [53, 54]. However, the importance of delivery method should be taken into account as direct needle injection may not be feasible or effective for liver tumors. Here, we have shown that stimulating the TME with a regionally delivered class C TLR9 agonist enhanced the anti-PD-1 antitumor effect for LM up to day 12 post first treatment.

While the data presented herein suggest that regional intravascular delivery of a class C TLR9 agonist can support control of LM directly and cause enhancement of systemic CPI effects via myeloid cell reprogramming, certain limitations should be considered. The murine model of PEDD™ recapitulates enhancement of flow and presure to LM, but does not involve use of the clinical device due to size constraints of the murine model. Although the data do support the ability of class C TLR9 agonist to reduce MDSC and favorably repolarize macrophages, we did not examine other immune cell populations that may also be contributing to the favorable effects of regional class C TLR9 agonist infusions.

In conclusion, our data provide evidence that regional delivery of class C TLR9 agonists are capable of altering the TME in LM, by eradicating MDSCs and favorably polarizing liver myeloid cells to blunt the impact of the highly immunosuppressive intrahepatic space on systemic CPI. Together, the present study provides evidence demonstrating favorable TME reprogramming through regional TLR9 agonist administration with PEDD, which was associated with deeper responses to systemic CPI for the treatment of LM.

## Supplementary information

Supplementary Figure Legend

Supplementary Figure 1

Supplementary Figure 2

Supplementary Figure 3

## Data Availability

The authors declare that the data supporting our findings are included in the paper and its supplementary information files.
