# Supplementary material for: Regional infusion of a class C TLR9 agonist enhances liver tumor microenvironment reprogramming and MDSC reduction to improve responsiveness to systemic checkpoint inhibition
Source: Cancer Gene Ther. 2022 Jun 14;29(12):1854–65. doi: 10.1038/s41417-022-00484-z (PMC9750861; doi:10.1038/s41417-022-00484-z)
Supplement: Supplementary file 1 — Supplementary Figure Legend [file 41417_2022_484_MOESM1_ESM.docx]

Supplementary Figure 1: Densitometric quantification of WB shown in Figure 1C

Densitometric analysis was performed and the ratio of (i) phospho/total NFκB, (ii) IL6/GAPDH and (iii) phospho/total STAT3 were quantified. Results are shown as mean + SEM. Students’ t-test was performed (*p <0.05, **p <0.01; n=6).

Supplementary Figure 2: Determination of TLR9-mediated effect on cytokine production

Human PBMCs were isolated from Donor 3 and 4 and were treated with increasing concentrations (0.04‑10 µM) SD101, ODN2395 along with ctrl ODN5328 (1 µM) for 48 h Supernatants were analyzed for (i) IL29, (ii) IFNα, (iii) IL6 and (iv) IL10 using Luminex assay.

Supplementary Figure 3: TLR9 is expressed in mouse LM-MDSCs

MDSCs were isolated from mouse LM using CD11b^+^Gr1^+^ negative selection method. Cells were treated with SD101 for 24 h. Isolated RNA was analyzed for TLR9 and IL10 by qRT-PCR. GAPDH was used as housekeeping gene control. MDSCs were isolated from 3 independent animals and mean ± SEM was plotted in the graph.
