# Supplementary figures and images for: Regional infusion of a class C TLR9 agonist enhances liver tumor microenvironment reprogramming and MDSC reduction to improve responsiveness to systemic checkpoint inhibition
Source: Cancer Gene Ther. 2022 Jun 14;29(12):1854–65. doi: 10.1038/s41417-022-00484-z (PMC9750861; doi:10.1038/s41417-022-00484-z)

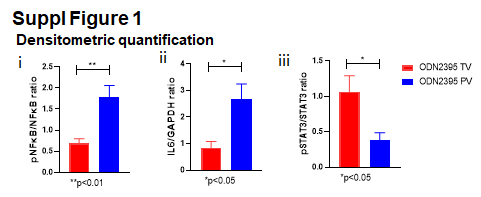

Supplement: Supplementary file 2 — Supplementary Figure 1 [file 41417_2022_484_MOESM2_ESM.tif]

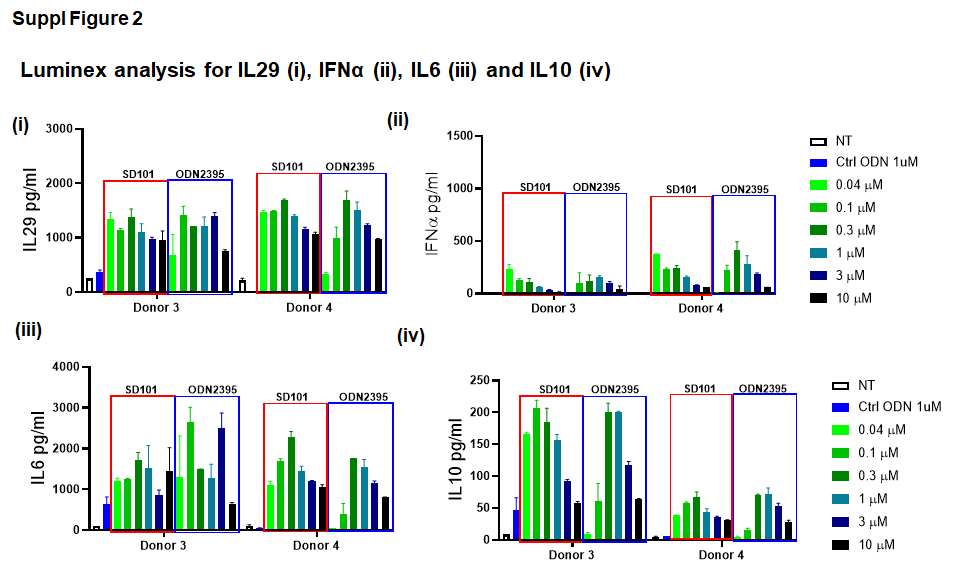

Supplement: Supplementary file 3 — Supplementary Figure 2 [file 41417_2022_484_MOESM3_ESM.tif]

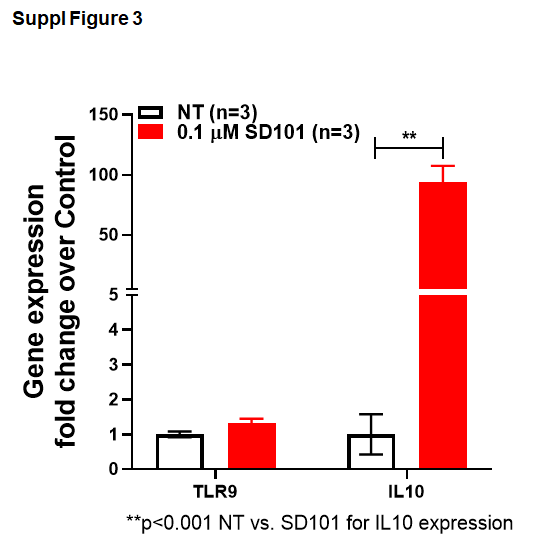

Supplement: Supplementary file 4 — Supplementary Figure 3 [file 41417_2022_484_MOESM4_ESM.tif]
